# Efficacy of PSMA PET-Guided Radiotherapy for Oligometastatic Castrate-Resistant Prostate Cancer

**DOI:** 10.3389/fonc.2021.664225

**Published:** 2021-04-19

**Authors:** Christoph Henkenberens, Thorsten Derlin, Frank Bengel, Tobias L. Ross, Markus A. Kuczyk, Frank A. Giordano, Gustavo R. Sarria, Leonard Christopher Schmeel, Hans Christiansen, Christoph A. J. von Klot

**Affiliations:** ^1^ Department of Radiotherapy and Special Oncology, Hannover Medical School, Hannover, Germany; ^2^ Department of Radiation Oncology, University Hospital Bonn, Bonn, Germany; ^3^ Department of Nuclear Medicine, Hannover Medical School, Hannover, Germany; ^4^ Department of Urology and Urologic Oncology, Hannover Medical School, Hannover, Germany

**Keywords:** PSMA, radiotherapy, castrate-resistant, oligometastases, metastasis-directed therapy

## Abstract

**Purpose:**

To assess the outcome of radiotherapy (RT) to all PSMA ligand positive metastases for patients with castrate-resistant prostate cancer (mCRPC).

**Patients and methods:**

A total of 42 patients developed oligometastatic mCRPC and received PSMA PET-guided RT of all metastases. The main outcome parameters were biochemical progression-free survival (bPFS), and second-line systemic treatment free survival (SST-FS).

**Results:**

A total of 141 PSMA ligand-positive metastases were irradiated. The median follow-up time was 39.0 months (12-58 months). During the follow-up five out of 42 (11.9%) patients died of progressive mPCa. Five out of 42 (11.9%) patients showed no biochemical responses and presented with a PSA level ≥10% of the baseline PSA at first PSA level measurement after RT and were classified as non-responders. The median PSA level before RT was 4.79 ng/mL (range, 0.4-46.1), which decreased significantly to a median PSA nadir level of 0.39 ng/mL (range, <0.07-32.8; p=0.002). The median PSA level at biochemical progression after PSMA ligand-based RT was 2.75 ng/mL (range, 0.27-53.0; p=0.24) and was not significantly different (p=0.29) from the median PSA level (4.79 ng/mL, range, 0.4-46.1) before the PSMA ligand-based RT. The median bPFS was 12.0 months after PSMA ligand PET-based RT (95% CI, 11.2-15.8) and the median SST-FS was 15.0 months (95% CI, 14.0-21.5).

**Conclusion:**

In well-informed and closely followed-up patients, PSMA PET-guided RT represents a viable treatment option for patients with oligometastatic mCRPC to delay further systemic therapies.

## Introduction

The cornerstone of treatment for metastatic castrate-resistant prostate cancer (mCRPC) is either cytotoxic chemotherapy, androgen biosynthesis inhibition (e.g. abiraterone), androgen receptor inhibition (enzalutamide), or radium-223. Androgen deprivation therapy (ADT) represents the column of systemic therapies, as most of the tumoral burden might remain sensitive to its effects. The escalation of systemic therapies is often associated with a negative impact on quality-of-life (QoL) (1). A small subgroup of patients with oligoprogression, defined as the development or progression of a limited number of lesions, might be controlled by radiotherapy as a metastasis-directed therapy (MDT) when targeting all lesions (2). These patients may continue on ADT for a defined period until further disease progression requires second-line systemic treatment (SST) (3). The recent introduction of prostate-specific membrane antigen (PSMA)-ligand positron emission tomography (PET) has substantially improved the diagnostic accuracy of staging at low prostate-specific antigen (PSA) levels (4–8). This technique yields further refined and well-monitored individualized radio-oncological treatment schemes which aim to improve PSA kinetics, prolong the progression-free survival and potentially defer the initiation of systemic therapies for patients with hormone-sensitive metastatic prostate cancer (mPCA) (9–14). Data on the feasibility and clinical outcome of MDT guided by PSMA-targeted imaging in mCRPC are limited.

Herein, we retrospectively assessed the outcomes of patients with mCRPC treated with PSMA PET-guided radiotherapy (RT) to all PET-positive metastases.

## Patients And Methods

We retrospectively assessed the clinical outcome of patients treated between June 2014 and May 2019 at a single institution for oligoprogressive PCa among ADT. These patients were classified as early mCRPC and received definitive PSMA PET-guided RT as MDT for all metastases. Criteria for mCRPC were either biochemical progression or radiologic progression according to EAU-ASTRO-SIOG Guidelines (1). No patient received additional systemic second-line treatment like docetaxel, novel androgen axis drug or any other drug. Oligometastatic disease was defined as ≤5 visceral or bone metastases. No limit on lymph node metastases was considered. The patients’ characteristics are summarized in **Table 1**.

**Table 1:** Patient characteristics (n = 42).

Characteristics	Median (range); n (%)
Age at PCa diagnosis	65.5 (49–84)
Initial PSA (ng/ml)	9.8 (3.7–84.5)
Primary therapy	
RPE alone	11 (26.2)
RPE +aRT	13 (31.0)
RPE + sRT	14 (33.3)
EBRT + temporary ADT	4 (9.5)
Initial T stage	
cT1c	5 (11.9)
pT2a,b	4 (8.1)
pT2c	13 (31.0)
pT3a	7 (16.7)
pT3b	11 (26.2)
pT4 a,b	0
unknown	2 (4.8)
Gleason-Score	
7a	8 (19.0)
7b	10 (23.8)
8	13 (31.0)
9	11 (26.2)
Initial N stage	
N0	30 (71.4)
N1	8 (19.0)
Surgical margins	
R0	34 (81.0)
R1	4 (9.5)
unknown	4 (9.5)
Initial risk group	
Low Risk	0
Intermediate Risk	9 (21.4)
High Risk	31 (73.8)
unknown	2 (4.8)
PSA nadir after definitive therapy (ng/ml)	0.07 (<0.07–5.2)
Interval (m) from definitive therapy to PSMA PET	76 (19–178)
PSA level at PSMA ligand PET imaging (ng/ml)	4.79 (0.4–46.1)
Patients with ADT at PSMA ligand PET imaging	42 (100%)
Median Duration of ADT at time of PSMA-PET imaging (m)	40.0 (12–180)
Median PSA dt at time of PSMA-PET imaging (m)	7.6 (3.6–50.5)

ADT, androgen deprivation therapy; aRT, adjuvant radiotherapy; dt, doubling time, EBRT, external beam radiation therapy; m, months; PCa, prostate cance;, PSMA ligand PET, prostate-specific membrane antigen ligand positron emission tomography; PSA, prostate-specific antigen; m, months; RP, radical prostatectomy; sRT, salvage radiotherapy.

### PET Imaging

Each patient underwent PET imaging with a ^68^Gallium-labeled PSMA ligand (15). Imaging acquisition was performed according to the joint EANM and SNMMI guideline (16). PSMA-ligand PET scans were acquired in conjunction with low-dose computed tomography (CT) on a dedicated PET/CT system (Siemens Biograph mCT 128 Flow; Siemens, Knoxville, TN) equipped with an extended field-of-view lutetium oxyorthosilicate PET component, a 128-slice spiral CT component, and a magnetically powered table optimized for continuous scanning. No intravenous contrast material was administered. All patients gave written informed consent before PSMA ligand PET/CT. A positive visual assessment of increased focal tracer uptake higher than the surrounding background activity was used as the criterion for malignancy (6).

### Radiotherapy Treatment

Patients with lymph node metastases or relapse in the prostatic fossa were treated with conventionally fractionated RT (CF-RT), and patients with bone metastases were treated with mild hypofractionated RT (HF-RT). In cases of lymph node metastases, the clinical target volume (CTV) encompassed the lymph drainage vessel to the next bifurcation or joint, excluding the whole ipsilateral lymphatic drainage. The prescribed dose was 50.0 Gray (Gy, single dose 2.0 Gy), followed by a sequential CF-RT boost of 10.0 Gy (single dose 2.0 Gy) to the lymph node metastases. Prostate bed relapses were treated with CF-RT doses of 70.0–74.0 Gy (single dose of 2.0 Gy). Bone metastases were treated with HF-RT at single doses of 2.5 Gy to a total of 45.0 Gy. The planning target volume (PTV) for lymph node metastases, bone metastases and local relapse in the prostatic fossa included the CTV plus a 10 mm safety margin in all directions, accounting for setup errors. Image guidance was conducted at least twice a week with megavoltage cone-beam CT. Visceral metastases were treated with image-guided stereotactic body radiation therapy (SBRT) to a total dose of 37.5 (single dose 12.5 Gy), prescribed to the 67% PTV marginal isodose. The PTV included the internal target volume (ITV) plus a 4 mm safety margin in all directions to account for setup errors.

### Follow-Up and Endpoints

All patients had periodic follow-up evaluations, which included PSA measurements every three months. Biochemically progressive disease after RT was defined as two consecutive increases in PSA levels from the nadir PSA level or a PSA level above baseline. Biochemical nonresponse was defined as a ≥10% PSA level elevation three months after RT, in comparison to the baseline PSA level at the time of PSMA ligand PET/CT scan before RT (9, 14). To assess the local failure patterns and rates, the PSMA PET/CT scans underwent a coregistration procedure with the RT treatment plans. Focally increased tracer uptake higher than the surrounding background within the PTV was classified as infield relapse. A second PSMA ligand PET/CT for the assessment of the pattern of relapse was available for 22 of 42 (52.4%) patients. Points of interest included the estimated biochemical progression-free survival (bPFS), second-line systemic treatment free survival (SST-FS), overall survival (OS) and toxicity rates. RT-associated toxicity was analyzed using the National Cancer Institute Common Terminology Criteria for Adverse Events (CTCAE) v4.0 (17).

### Statistical Analysis

The statistical analysis was performed with The Jamovi Project (2020), Jamovi (Version 1.6.3) for Windows. Retrieved from https://www.jamovi.org. The paired Student’s *t*-test to compare pre-RT with post-RT parametric parameters and the Wilcoxon signed-rank for non-normally distributed data were applied. The estimated survival rates were calculated using the Kaplan–Meier method. Factors for RT treatment failure were analyzed with the log-rank test in univariate analyses, and significant factors were further assessed with multivariate analyses using a binominal logistic regression method to identify independent variables. P-values <0.05 were considered statistically significant. Graphical presentations of the patterns of progression were created using a free software for statistical computing and graphics (R Version 3.0.3).

### Ethics Statement

This retrospective study was approved by the local institutional review board (IRB), aligned with the principles of the Declaration of Helsinki. All cases were discussed and approved for RT by the multidisciplinary uro-oncologic board. Informed consent was obtained prior to patients’ participation.

## Results

### Result of PSMA Ligand PET Staging and Therapy for Metastases

Data from a total of 42 patients were analyzed. One hundred and forty-one PSMA ligand-positive metastases were detected and treated with RT: Pelvic nodal metastases accounted for 37.6% (53/141), while 18.4% (26/141) allocated in paraaortic node metastases, 7.8% (11/141) in distant lymph node metastases, 28.4% (40/141) in bone metastases, 1.4% (2/141) in visceral metastases, and 6.4% (9/141) in local prostatic fossa relapses. Regarding distribution patterns, 30.9% of patients (13/42) developed only nodal metastases and 23.8% (10/42) only bone metastases. Additionally, 21.4% (9/42) of patients presented both lymph node and bone metastases, 4.8% (2/42) visceral metastases, 4.8% (2/42) relapse in the prostatic fossa, 4.8% (2/42) relapse in the prostatic fossa and bone metastases, and 9.5% (4/42) relapse in the prostatic fossa and lymph node metastases.

### Patterns of Progression and Patient Outcomes

**Table 2** summarizes the results for the 22 patients who had a first PSMA ligand PET prior to RT and a restaging PSMA ligand PET after biochemical progression occurred.

**Table 2:** Results of first PSMA ligand PET staging prior to PSMA PET-guided radiotherapy and second PSMA ligand PET for restaging after biochemical progression (n = 22).

	First PSMA ligand PET prior to RT	Second PSMA ligand PET after biochemical progression
	N (%)	N (%), p value
No. of PSMA-ligand positive lesions	73 (100)	100 (100); 0.08
Total no. of LNs	54 (73.9)	54 (54.0); 0.32
Pelvic LNs	32 (43.8)	18 (18.0); 0.11
Periaortic/interaortocaval LNs	18 (24.7)	29 (29.0); 0.14
Distant LNs	4 (5.5)	7 (7.0):0.29
Total no. of bone metastases	17 (23.3)	44 (44.0); 0.03
Pelvic bone	12 (16.4)	11 (11.0); 0.59
Extrapelvic bone	5 (5.4)	33 (33.0); 0.01
Prostatic fossa	1 (1.4)	1 (1.0); 0.50
Total no. of visceral metastases	1 (1.4)	1 (1.0); 0.91
	Median (range)	Median (range)
No. irradiated metastases	3 (1–11)	3 (1–13)

LNs, lymph node metastases; PSMA ligand PET, prostate-specific membrane antigen ligand positron emission tomography.

**Figure 1:**
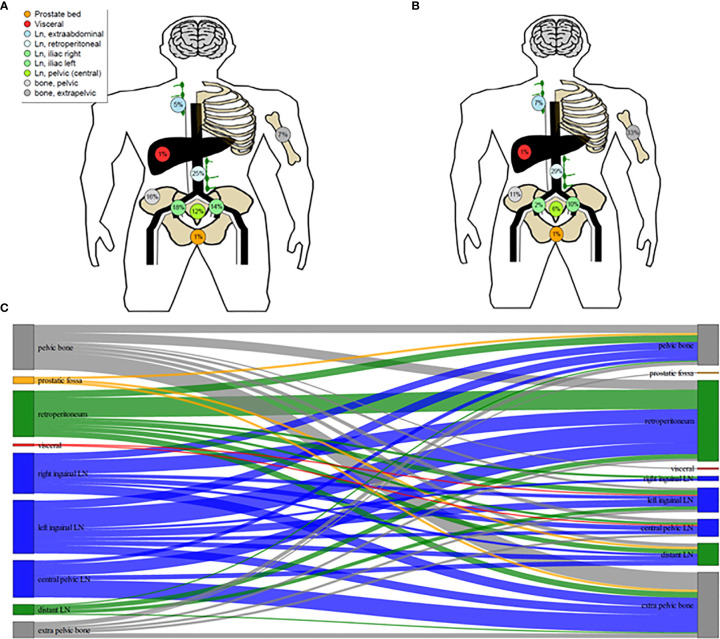
Schematic illustration of ^68^Ga-PSMA ligand PET/CT distribution of metastases of oligoprogressive prostate cancer under androgen deprivation therapy prior to radiotherapy (RT) **(A)** and distrubution of metastases at further progression after PSMA-guided RT **(B)**. Migration of Metastases **(C)**.

The anatomical distributions and migration of metastases are shown in **Figures 1A–C**. Analysis of the RT treatment plans and the second PSMA ligand PET/CT scans resulted in an infield relapse rate of 2.7% (2/73). The two infield relapses occurred in the right iliac lymph nodes and in the spine.

**Figure 2:**
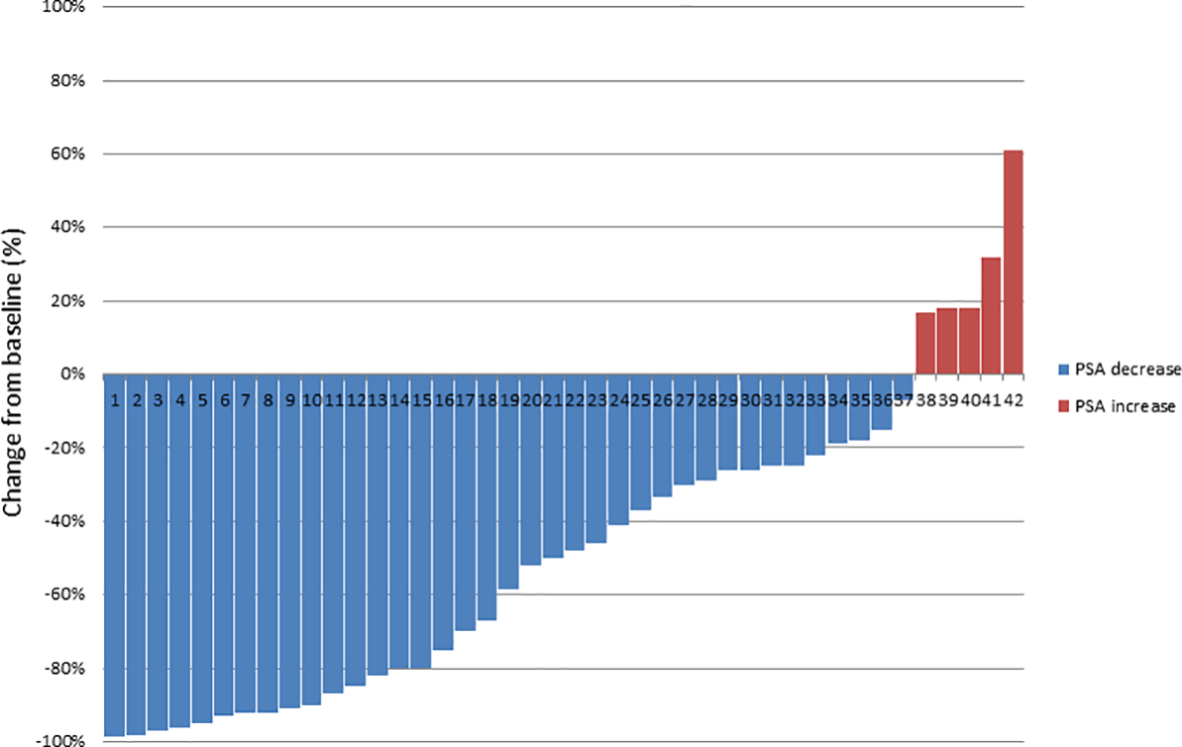
Waterfall plot of best PSA response, based on maximal percentage of PSA change.

The median follow-up time was 39.0 months (12–58). During the follow-up five (11.9%) patients died of progressive mPCa; in addition, five (11.9%) patients showed no biochemical responses and were classified as non-responders, as a PSA level rise ≥10% above the baseline after first post-RT measurement was evidenced. The median PSA level prior to RT was 4.79 ng/ml (0.4–46.1), which decreased significantly to a median PSA nadir level of 0.39 ng/ml (<0.07–32.8; p = 0.002) following RT. **Figure 2** shows a waterfall plot of the PSA response. The median PSA level at biochemical progression after PSMA PET-guided RT was 2.75 ng/ml (0.27–53.0; p = 0.24), and thus not significantly different (p = 0.29) from the median PSA level (4.79 ng/ml, 0.4–46.1) before the PSMA PET-guided RT. Additionally, 14.3% (6/42) of patients did not show biochemical progression at their last follow-up. Concerning the 36 patients with biochemical progression, two (5.5%) patients declined SST and chose observation; the patient with the infield lymph node relapse received salvage surgery (1/36, 2.8%). Nine patients (25.0%) received a second PSMA PET-guided RT to all new metastases and SST when further biochemical progression after second PSMA PET-guided RT occurred. Furthermore, 24 patients (66.7%) received SST.

**Figure 3:**
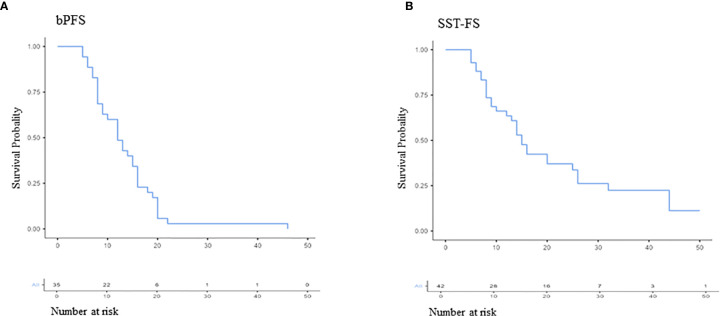
Kaplan–Meier curves of biochemical progression-free survival (bPFS; **A**) and second-line systemic treatment free survival (SST-FS; **B**).

The median bPFS was 12.0 months after PSMA ligand PET-based RT (95% CI, 11.2–15.8; **Figure 3A**), and the median SST-FS was 15.0 months (95% CI, 14.0–21.5; **Figure 3B**). None of the analyzed parameters for bPFS was statistically significant in univariate analyses. The significant parameters in univariate analyses for SST-FS were initial PSA level >10 ng/ml (p = 0.04), the number of irradiated metastases (p = 0.02) and the peak standardized uptake value (SUVpeak). None of the significant parameters reached significance in multivariate analyses. **Table 3** shows the detailed results of the uni- and multivariate analyses for bPFS and SST-FS.

**Table 3:** Results of first PSMA ligand PET staging prior to PSMA PET-guided radiotherapy and second PSMA ligand PET for restaging after biochemical progression (n = 21).

	SST-FS		bPFS	
	Univariate analysis	Multivariate analysis	Univariate analysis	Multivariate analysis
	*p*-value (OR, 95% CI)	*p*-value (OR, 95% CI)	*p*-value (OR, 95% CI)	*p*-value (OR, 95% CI)
Initial T stage (≤T2 vs. ≥T3)	0.35 (1.31, 0.67–2.97)	–	0.26 (1.51, 0.74–3.01)	–
Initial N stage (N0 vs. N1)	0.36 (1.63, 0.52–5.75)	–	0.84 (1.02, 0.46–2.26)	–
Initial PSA level in ng/ml	0.69 (1.05, 0.97–1.03)	–	0.39 (1.08, 0.98–1.03)	–
Initial PSA level >20 ng/ml	0.58 (1.12, 0.54–2.98)	–	0.41 (1.34, 0.36–1.52)	–
Initial PSA level >10 ng/ml	0.04 (2.16, 1.20–4.54)	0.98 (0.27–14.60)	0.28 (1.53, 0.69–3.38)	–
PSA nadir after RP	0.40 (1.03, 0.62–1.23)	–	0.46 (0.95, 0.82–1.09)	–
Gleason score	0.29 (1.05, 0.36–3.16)	–	0.86 (1.03, 0.72–1.47)	–
Initial high risk	0.18 (1.63, 0.79–3.36)	–	0.89 (0.95, 0.48–1.88)	–
Duration of ADT	0.22 (1.07, 0.96–1.20)		0.13 (1.19, 0.97–1.20)	
PSA-dt at PSMA PET	0.61 (1.02, 0.97–1.05)	–	0.62 (1.01, 0.96–1.06)	–
PSA at PSMA PET	0.24 (1.03 0.98–1.06)	–	0.95 (1.00, 0.97–1.03)	–
SUVpeak	0.02 (1.21, 1.05–1.31)	0.48 (1.03 0.96–1.10)	0.12 (1.13, 0.87–1.15)	–
No. of irradiated metastases	0.02 (1.26, 1.04–1.40)	0.08 (1.9, 0.93–4.06)	0.14 (1.17, 0.84–1.38)	–
LN metastases only	0.23 (0.64, 0.3–1.35)	–	0.62 (0.90, 0.45–1.81)	–
Extrapelvic disease (LNs and/or bone)	0.53 (1.2, 0.38–1.56)	–	0.61 (1.13, 0.41–1.69)	–

dt, doubling time; LN, lymph node; PSA, prostate-specific antigen; RP, radical prostatectomy; SUV, standardized uptake value.

### Toxicity

Acute grade III toxicity was not observed; 4.8% (2/42) of patients developed grade II acute gastrointestinal side effects. Late grade I gastrointestinal toxicity occurred in 2.4% (1/42) of patients. Late grade ≥II toxicities were not observed.

## Discussion

The implementation of PSMA ligand-based imaging has substantially improved the diagnostic accuracy of detecting metastatic PCa at low PSA levels (6–8). Although large randomized prospective phase III studies are lacking (12–14, 18), there is a strong consensus among experts that MDT is considered a viable treatment option for well-selected patients, mostly with oligorecurrent PCa (19). However, these trials investigated the potential of MDT to delay the initiation of ADT for asymptomatic hormone-naive metastatic PCa (13, 14, 18). Patients undergoing ADT with increasing PSA levels and detection of a limited number of metastases in PSMA-ligand PET are regarded as early mCRPC and information on MDT in this setting is scarce. Usually, biochemical progression is the trigger for staging imaging, and iconographic progression (in some patients only biochemical progression) is the indication for SST (1). Further systemic therapies are associated with both non-negligible toxicities and increased healthcare expenditures (20). However, a small subgroup of patients might not benefit from SST as the vast majority of disease is still controlled through the ongoing systemic therapy (21). The biological rationale encompasses the evolution of a few cell-line subpopulations within different metastases under the selection pressure of ADT towards a more aggressive phenotype, driving the ominous course of the disease (22). Eradicating these lesions might delay the initiation of SST. Additionally, those large trials which investigated either docetaxel, abiraterone or enzalutamide as SST for mCRPC did not include a sufficient number of participants with low PSA-levels and low tumor burden. It remains, therefore, difficult to draw precise conclusions on the exact benefit and optimal timing of SST for these patients (23–25).

Based on the biological rationale we retrospectively assessed the clinical outcome of patients with early mCRPC who received RT as MDT to all PSMA positive lesions, and found that the decreased PSA levels lead to a subsequent delay of further systemic therapies. We observed that RT targeting all metastases detected by PSMA-ligand PET postponed the second-line systemic therapies for a median of 15 months with only negligible RT-related side effects. Other reports on MDT for mCRPC have reported similar SST-FS (9, 26–29). However, their outcomes mainly report on patients with both metastatic hormone-sensitive and castration-resistant PCa with limited information on the clinical outcome of patients with mCRPC (27, 28). Furthermore, some patients received RT plus SST which limits a comparison with our results (26). Other retrospective studies assessed the benefit of cytoreductive RT for patients with mCRPC plus abiraterone (30) or for patients with progressive disease among abiraterone or enzalutamide, showing that RT might delay disease progression in both clinical scenarios (31–33). Additionally, there are no data on PSMA-ligand PET for staging purposes prior to RT as MDT for mCRPC.

To our best knowledge, we present the first data including a more homogeneous mCRPC patient cohort staged with PSMA-ligand PET/CT, who received RT to all metastases as MDT to delay the initiation of SST. The observed median SST-FS of 15 months is encouraging, although we found no significant clinical parameter influencing the observed outcome (bPFS and SST-FS). This suggests that clinical parameter do not drive the clinical course, leading to a demand for molecular biomarkers in the presented clinical study (34, 35).

Some limitations to this study should be acknowledged. Its retrospective nature has inherent limitations and might have incurred in selection bias, although the study cohort had a strict follow-up schedule and a PSMA PET-based staging protocol prior to RT was performed fewer metastases should thus have been missed to diagnosis as compared to conventional imaging or choline PET techniques (6–8, 20, 36). There is controversy about the radiation dose, field size, and elective node irradiation when PSMA ligand PET is used for MDT of oligorecurrent mPCa. Data from the choline PET era confirmed that choline PET underestimated the extent of lymph node metastases (37), which is reflected by the fact that approximately two out of three patients treated with SBRT for pelvic lymph node metastases relapsed with lymph node metastases (38, 39), leading to a higher relapse rate than that after elective node irradiation (ENI), although the relapse rate concerning bone and visceral metastases seems to be comparable between SBRT and ENI (40). Additionally the optimal definition of biochemical progression for this emerging clinical scenario does not exist. The PCWG2 (prostate cancer clinical trials working group) definitions for mCRPC were designed to measure outcomes for drug trials that evaluate systemic treatment for mCRPC and “high” PSA levels to improve the alignment of clinical research and practice (41). In our patient cohort the median PSA-level of 4.79 ng/ml was significantly lower than the PSA level in any drug trial on mCRPC. Additionally the PCWG2 definition does not include any information about local therapies. So we used the above mentioned more conservative definition based upon our previous reports, where radiographic clinical progression using PSMA PET showed high concordance with PSA increase (9, 14) allowing refined and well-monitored personalizedradio-oncological treatment concepts. The sample size of 42 patients limited the statistical power. Moreover, the study included a selected cohort with mainly baseline high-risk PCa. In this sense, caution is advised when translating the observed results to clinical practice.

Taken together, the observed clinical results are robust and contribute significantly to set the basis of PSMA guided-RT as MDT in a rapidly evolving clinical field.

## Conclusion

PSMA PET-guided RT to all enhancing metastases in the mCRPC setting delayed systemic therapies without major toxicity and represents a promising treatment option. Prospective evaluation is warranted to confirm these findings.

## Data Availability Statement

The raw data supporting the conclusions of this article will be made available by the authors, without undue reservation.

## Ethics Statement

The studies involving human participants were reviewed and approved by Medical School Hannover, Ethics Committee. The patients/participants provided their written informed consent to participate in this study.

## Author Contributions

CH and CK contributed to conception and design of the study. CH and HC enrolled patients. TR, TD, and FB were responsible for conduction and reporting of the PSMA-PET/CTs. MK, FG, GS, and LS wrote and revised sections of the manuscript. All authors contributed to the article and approved the submitted version.

## Conflict of Interest

The authors declare that the research was conducted in the absence of any commercial or financial relationships that could be construed as a potential conflict of interest.
