# Supplementary material for: Efficacy of PSMA PET-Guided Radiotherapy for Oligometastatic Castrate-Resistant Prostate Cancer
Source: Front Oncol. 2021 Apr 19;11:664225. doi: 10.3389/fonc.2021.664225 (PMC8089391; doi:10.3389/fonc.2021.664225)
Supplement: Supplementary file 1 [file Table_1.docx]

**Toxicity**

|  | Grade I acute | Grade II acute | Grad I late |
| --- | --- | --- | --- |
| Gastrointestinal | 14.3% (6/42) | 4.8% (2/42) | 2.4% (1/42 |
| Genitourinary | 4.8% (2/42) |  |  |
| Fatigue | 16.7% (7/42) |  | 9.5% (4/42) |
| Other | 7.9% (3/42) |  |  |

Acute Toxicities according to CTCAE (Common Terminology Criteria for Adverse Events)

Late Toxicities according to LENT-SOMA (Late effects Normal Tissues-Subjective, Objective, Management, Analytic) scales
